# Vaccine Composition Formulated with a Novel *Lactobacillus*-Derived Exopolysaccharides Adjuvant Provided High Protection against *Staphylococcus aureus*

**DOI:** 10.3390/vaccines9070775

**Published:** 2021-07-12

**Authors:** Haochi Zhang, Na Pan, Cheng Ma, Bohui Liu, Lei Xiu, He Tong, Shouxin Sheng, Yanchen Liang, Haotian Li, Fangfei Ma, Xuemei Bao, Wei Hu, Xiao Wang

**Affiliations:** State Key Laboratory of Reproductive Regulation and Breeding of Grassland Livestock, Inner Mongolia University, Hohhot 010070, China; 21908028@mail.imu.edu.cn (H.Z.); 22008032@mail.imu.edu.cn (N.P.); 31808081@mail.imu.edu.cn (C.M.); 31808136@mail.imu.edu.cn (B.L.); 21708020@mail.imu.edu.cn (L.X.); 21908040@mail.imu.edu.cn (H.T.); shouxins1230@mail.imu.edu.cn (S.S.); yanchenliang@mail.imu.edu.cn (Y.L.); 31908093@mail.imu.edu.cn (H.L.); 31908098@mail.imu.edu.cn (F.M.); 31908096@mail.imu.edu.cn (X.B.); huw@imu.edu.cn (W.H.)

**Keywords:** exopolysaccharides, vaccine, *S. aureus*, γδ T cells, IL-17A

## Abstract

A vaccine that effectively targets methicillin-resistant *Staphylococcus aureus* (MRSA) is urgently needed, and has been the focus of studies by numerous research groups, but with limited success to date. Recently, our team found that exopolysaccharides derived from probiotic *Lactobacillus* *casei* strain WXD030 as an adjuvant-formulated OVA could upregulate IFN-γ and IL-17 expression in CD4^+^ T cells. In this study, we developed a vaccine (termed rMntC-EPS) composed of *S. aureus* antigen MntC and *Lactobacillus* *casei* exopolysaccharides, which conferred high levels of protection against *S. aureus* infection. Methods: Six–eight-week-old female mice were vaccinated with purified rMntC-EPS30. The immune protection function of rMntC-EPS30 was assessed by the protective effect of rMntC-EPS30 to *S. aureus*-induced pulmonary and cutaneous infection in mice, bacterial loads and H&E in injury site, and ELISA for inflammation-related cytokines. The protective mechanism of rMntC-EPS30 was assessed by ELISA for IgG in serum, cytokines in the spleen and lungs of vaccinated mice. In addition, flow cytometry was used for analyzing cellular immune response induced by rMntC-EPS30. For confirmation of our findings, three kinds of mice were used in this study: IL-17A knockout mice, IFN-γ knockout mice and TCRγ/δ knockout mice. Results: rMntC-EPS30 conferred up to 90% protection against *S. aureus* pulmonary infection and significantly reduced the abscess size in the *S. aureus* cutaneous model, with clearance of the pathogen. The rMntC-EPS vaccine could induce superior humoral immunity as well as significantly increase IL-17A and IFN-γ production. In addition, we found that rMntC-EPS vaccination induced robust Th 17/γδ T 17 primary and recall responses. Interestingly, this protective effect was distinctly reduced in the IL-17A knockout mice but not in IFN-γ knockout mice. Moreover, in TCRγ/δ knockout mice, rMntC-EPS vaccination neither increased IL-17A secretion nor provided effective protection against *S. aureus* infection. Conclusions: These data demonstrated that the rMntC formulated with a novel *Lactobacillus*-derived Exopolysaccharides adjuvant provided high protection against *Staphylococcus aureus*. The rMntC-EPS vaccine induced γδ T cells and IL-17A might play substantial roles in anti-*S. aureus* immunity. Our findings provided direct evidence that rMntC-EPS vaccine is a promising candidate for future clinical application against *S. aureus*-induced pulmonary and cutaneous infection.

## 1. Introduction

*Staphylococcus aureus* is a gram-positive bacterium with a complex pathogenesis mechanism. *S. aureus*-induced pneumonia accounts for 20–50% of nosocomial pneumonia cases and ~25% of cases of community-acquired pneumonia, which causes severe pulmonary infection, with high morbidity and mortality [1,2]. *S. aureus* skin infections have been an increasingly serious public health problem in recent decades. Incidence of skin and soft tissue infections in the U.S. rose by ~40% from 2000 to 2012, and cost of treatments rose from USD 4.8 to USD 15.0 billion [3]. Methicillin-resistant *S. aureus* (MRSA) strains have appeared and become increasingly resistant to multiple antibiotics [4]; therefore, an effective prophylactic vaccine that specifically targets *S. aureus* is urgently needed. To date, attempted vaccine development has focused mainly on stimulating antibody response. However, StaphVAX and IsdB did not provide effective protection against *S. aureus* bacteremia in phase III clinical trials, most likely because of overemphasis on humoral immune responses, suggesting that the design of next-generation vaccines should focus on targeted T cell responses [5,6].

Th1 and Th17 cell-mediated immunity plays a key role in resistance against *S. aureus* infection [7,8,9]. A vaccine targeting *S. aureus*-induced pneumonia significantly reduced bacterial load when i.p. injected; this effect was mediated by IFN-γ and IL-17 produced by CD4^+^ T cells [10]. H. Ishigame’s group observed that CD4^+^ T cells produced IL-17 in response to *S. aureus*-induced skin infection, and that bacterial load at infection site was higher in IL-17-deficient mice, indicating the importance of Th17 cells in clearance of local infections [11]. M. Holley and T. Kielian reported that Th cells helped coordinate the response of phagocytic effector cells to clear the infection, but did not directly target bacteria or infected cells. Macrophages and cytotoxic T cells were activated by IFN-γ, while IL-17 recruited neutrophils to infection sites [12]. CD8^+^ T cells, another adaptive T cell immune system, also produced IFN-γ and IL-17 and promoted resistance to *S. aureus* infection [13,14]. Roles of CD4^+^ and CD8^+^ T cells, and of IFN-γ and IL-17 produced by these cells, have been extensively studied in regard to resistance to *S. aureus* infection. These cells and the cytokines produced are considered to have strong potential as vaccine targets; however, attempted clinical applications have had limited success. In actuality, these cytokines are also produced by other innate immune cells, including γδ T cells, innate lymphoid cells (ILCs) and mucosal-associated invariant T (MAIT) cells; this ability is not unique to CD4^+^ and CD8^+^ T cells. T cells bearing γδ T cell receptor (TCR) (γδ T cells), a subset of innate immune lymphocytes, have key roles in mucosal host defenses against *S. aureus* infection and in regulating initial immune response to lung and skin pathogens by modulating the recruitment of neutrophils, dendritic cells and macrophages [15,16,17]. Because γδ T cells promote resistance to *S. aureus* infection by producing IL-17, which recruits neutrophils to infection sites for bacterial clearance, there is presumably strong potential for the development of vaccine therapy based on activation of γδ T cells [18,19,20]. Again, however, the attempted generation of vaccines that promote resistance to *S. aureus* infection based on the γδ T17 pathway have been largely unsuccessful.

Adjuvants activate innate immune system and also generate cytokine milieus that modulate vaccine-induced Th cell differentiation [21,22]. Vaccine-induced immune responses are clearly affected by administration route and adjuvant selection. Several potential adjuvants, including Aluminum adjuvant (Alum), Complete Freund’s Adjuvant (CFA), the cholera toxin B subunit (CTB), the *Escherichia coli*–derived heat-labile enterotoxin, α-galactosylceramide (αGalCer, a CD1 ligand), CpG-containing oligodeoxynucleotides (CpG) and the double-stranded RNA analog polyinosinic-polycytidylic acid (poly(I:C)), have been identified and extensively studied. Despite showing promise in terms of immune-stimulating activity, these adjuvants have yet to be licensed for human use, in large part because of safety concerns linked to local or systemic toxicity and reactogenicity [23]. Receptor agonist–based adjuvants, such as CpG (a toll-like receptor (TLR) 9 ligand) and poly(I:C) (a TLR3 ligand), have well-defined mechanisms of action. Other adjuvants, however, including aluminum salts, do not require TLR signaling for immune activation, implying the existence of distinct adjuvant-elicited immune activation pathways [24].

Host mucosal tissues such as gut and skin are cohabitated. The gut commensal microbiota is the most characterized microbial community in mammals, showing critical roles in gut immune development and gut physiology. Although it was historically thought to be sterile in health, the lower respiratory tract has been shown to contain a lung microbiome by recent culture-independent techniques such as high-throughput sequencing with symbiotic microbiota. Previous study proved that the lung commensal microbes, *L. murinus*, provides a barrier against pneumococcal colonization in a respiratory dysbiosis model after an influenza A virus infection, when added therapeutically. We previously demonstrated that exopolysaccharides of *Lactobacillus* *casei* upregulated IFN-γ and IL-17 expression in CD4^+^ T cells more strongly than Alum when formulated with OVA, and was shown to be clinically safe [25]. In this study, we developed a new vaccine, termed rMntC-EPS30, composed of *S. aureus* antigen MntC and *Lactobacillus* *casei* exopolysaccharides. *S. aureus* manganese transport protein C (MntC) has a site that can directly combine with Mn2^+^ to bind manganese, and in animal models of *S. aureus* systemic infection, MntC has been found to be highly expressed on the surface of the cell membrane, and is proven to be conserved across the staphylococcal species group.

We describe here that the new vaccine, rMntC-EPS30, induced γδ T cells and IL-17A, which played a substantial role in anti-*S. aureus* immunity. Efficacy of rMntC-EPS30 was comprehensively evaluated using two mouse models suitable for accurately monitoring progression of several diseases, including pneumonia and skin infection. These results suggested a key strategy for establishing next-generation *S. aureus* vaccines, including novel adjuvants.

## 2. Materials and Methods

### 2.1. Experimental Animals and Ethics Statement

Specific-pathogen-free (SPF) female C57BL/6 mice, age 6–8 wk, were purchased from Beijing Vital River Laboratory Animal Technology Co. (Beijing, China). IFN-γ-deficient mice (JAX Stock number: 002116), γδ T cell-deficient mice (JAX Stock number: 003288) and IL-17A-deficient mice (NCBI Stock number: 16171) on C57BL/6N background were kindly donated by Dr. Z. Yin (College of Life Sciences, Jinan University, Guangzhou, China). All animal-related experimental protocols applied in this study were conducted under the standards of the Ethics Committee of Inner Mongolia Medical University (SCXK2016-0001).

### 2.2. Bacterial Strains

*L. casei* WXD030 was isolated from traditional dairy products in Inner Mongolia, China, and maintained in our laboratory. It was identified as *L. casei* on the basis of physiological characteristics and 16S rDNA sequence analysis [25].

*S*. *aureus* strains USA300 (TCH 1516), 27,543 (NCDO 1499), 12,600 (NCTC 8532), and Newman (NCTC 10833) were purchased from from BeNa Culture Collection (Beijing, China).

### 2.3. Extraction and Purification of EPS30

*L. casei* WXD030 EPS were extracted by the method of Ai et al. [26] with slight modification. Crude EPS were purified using DEAE-Sepharose Fast Flow column, and carbohydrate content of the eluent was determined by phenol-sulfuric acid method with glucose as standard [27,28]. A polysaccharide fraction, termed EPS30, was obtained by elution of Tris-HCl buffer with linear NaCl concentration gradient, and further purified on Sepharose CL-6B gel column. Eluent containing EPS was detected, collected, dialyzed against deionized water for 2 d at 4 °C and lyophilized.

### 2.4. Expression Analysis of Recombinant MntC

*mntC* gene sequence (930 bp) was retrieved from GenBank database, and amplified by polymerase chain reaction (PCR) from genomic DNA of *S. aureus* strains 27,543, 12,600, USA300, and Newman using primers F: 5′-ATGAAAAAATTAGTACCTTTATTAT-3′/R: 5′-TTATTTCATGCTTCCGTGTACAGTT-3′ (Figure S1A). PCR products were cloned into pEASY-Blunt E1 expression vector with N-terminal 6-histidine-tag (His-tag), and transformed into *Escherichia coli* BL21(DE3) (TransGen Biotech, Beijing, China). Protein expression was induced by adding isopropyl β-D-1-thiogalactopyranoside (IPTG) to final concentration 1 mM, and cultures were grown at 37 °C with shaking (200 rpm) for 12 h. rMntC antigen expression was evaluated in vitro by Western blotting analysis (Figure S1B).

Cells were harvested by centrifugation at 8000× *g* for 15 min at 4 °C. Supernatant was carefully removed and applied to Ni-NTA agarose column (TransGen Biotech, Beijing, China). Expressed rMntC protein was estimated as 34 kDa on the basis of SDS-PAGE analysis and gel filtration chromatography (Figure S1C–E).

### 2.5. Vaccination

Purified rMntC or rGapC was diluted in PBS and mixed 1:1 for EPS30 or emulsified 1:1 for Alum or CFA. For murine pneumonia model, wild-type (WT), IFN-γ-deficient, IL-17A-deficient and γδ T cell-deficient mice were vaccinated by subcutaneous (s.c.) route (volume 200 μL containing 50 μg rMntC and 40 μg EPS30) in first vaccination. In boost immunization, mice were vaccinated by intranasally route (volume 40 μL containing rMntC or rGapC and EPS30 amounts as above). Two vaccinations were given at 2 wk interval. For murine skin infection model, two vaccinations were given at 2 wk interval by s.c. route (volume 200 μL containing rMntC and EPS30 amounts as above).

### 2.6. Murine Pneumonia Model

Vaccinated mice (female WT, C57BL/6, IFN-γ-deficient, IL-17A-deficient and γδ T cell-deficient) were injected with 180 μL 4% chloral hydrate. Mice were then anesthetized, inoculated with 40 μL bacterial slurry (5 × 10^9^ CFUs of *S. aureus* USA300) via left nostril and observed continuously for 48 h [29]. A total of 40 μL bacterial slurry (5 × 10^8^ CFUs of *S. aureus* USA300) was inoculated via left nostril, and numbers of bacteria in organs at 24 and 72 h were counted by preparing organ homogenates in PBS and plating on tryptic soy agar (BD Diagnosis System, New Jersey, USA). Colonies were counted after 24 h incubation at 37 °C. Levels of inflammatory cytokines in lung were determined by collecting lung homogenates at 24 and 72 h and analyzing TNF-α, IL-1β, IL-6 and IL-10 with Mouse ELISA Kits (R&D Systems, Shanghai, China) as per manufacturer’s instructions. Left lobes of lung were removed at 24 and 72 h, and tissues were fixed, paraffin-embedded, sectioned (4–6 μm) and stained with hematoxylin and eosin (H&E).

### 2.7. Murine Skin Infection Model

Female mice were anesthetized, inoculated by dorsal s.c. injection with 5 × 10^8^ CFUs of *S. aureus* USA300 in 50 μL, and monitored daily for 14 days for mass and abscess formation. Sizes of abscesses and associated overlying dermonecrotic lesions were determined by a standard equation: Area (A) = (π/2) × length × width [30]. For counting of *S. aureus* CFUs in skin abscess lesions, animals were euthanized 1 day after inoculation, abscesses were removed and homogenized in PBS and CFUs were counted by plating serially diluted samples on tryptic soy agar at 37 °C and growing for 24 h. Skin tissues were fixed, paraffin-embedded, sectioned (4–6 μm) and stained with H&E.

### 2.8. ELISA for Specific Antibodies

Blood samples (12 mice per group) were collected from the retinal vein on days 14 and 21 after first vaccination, stored at 37 °C for 2 h and centrifuged at 3000 rpm for 20 min to isolate serum. Serum antibodies titers including IgG, IgG1, IgG2a and IgG2b were measured by ELISA. Endpoint titer was defined as the highest dilution having OD value higher than cut-off value (i.e., 2.1-fold higher than negative control signal). Base 2 logarithms (log2) were used for data involving extremely high titers, and for graphical presentation of data.

### 2.9. Cytokine Determination

Animals (5 mice per group) were sacrificed on day 7 following boost vaccination. Briefly, lung and spleen tissues were perfused with 5 mL PBS containing 0.5% bovine serum albumin (BSA) and 2 mM EDTA to obtain single-cell suspensions. The cell suspensions were firstly depleted of red cells and then CD3^+^ T cells were isolated using a CD3 MicroBead Kit for mice (Miltenyi Biotec, Berlin, Germany) [25]. Isolated CD3^+^ T cells were incubated in 12-well plates at 5 × 10^4^ cells/well in a 5% CO_2_ incubator and stimulated with rMntC (10 µg/mL), and supernatants were collected after 72 h for ELISA analysis of IFN-γ, IL-4 and IL-17A levels.

### 2.10. Intracellular Cytokine Staining

Animals (5 mice per group) were sacrificed at 12 h after first vaccination, and on day 7 after boost vaccination, T cells from the spleen were prepared, and surface staining with anti-APC-CD3, -FITC-CD4, -FITC-CD8 or -PE-Cy7-TCRγ/δ antibody at 1:100 dilution was performed for 30 min at 4 °C. Cells were fixed and permeabilized with 0.1% saponin, and stained with intracellular cytokine PE-IL-17A. ~1 × 10^6^ events were acquired on FACS, and data were analyzed using De Novo software program. In other experiments, animals (5 mice per group) were sacrificed at 24 h post-infection, T cells from the lung and spleen were prepared, and IL-17A-secreting CD4^+^, CD8^+^, and γδ T cells were analyzed by a similar procedure as above.

### 2.11. Statistical Analysis

All experiments were performed in triplicate. GraphPad software program was used for data processing and analysis. Significance of differences with *p* < 0.05 was determined by analysis of variance (ANOVA). Differences among three or more groups were analyzed by one- or two-way ANOVA multiple comparisons. Significance of differences was classified as * *p* < 0.05, ** *p* < 0.01, *** *p* < 0.001 or n.s. (not significant).

## 3. Results

### 3.1. rMntC-EPS30 Vaccination Provided High Protection against S. aureus-Induced Pneumonia

**Figure 1:**
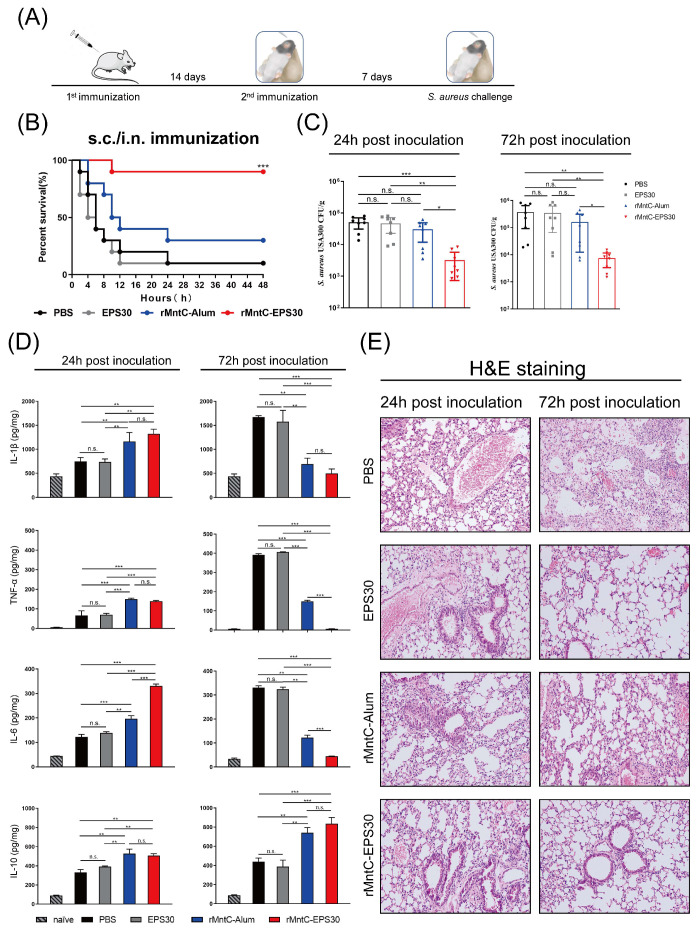
rMntC-EPS30 vaccination effectively promoted resistance to *S. aureus*-induced pneumonia. (**A**) Timeline of vaccination and *S. aureus*-induced pneumonia model. (**B**) Mice were inoculated with 40 μL bacterial slurry (5 × 10^9^ CFUs of *S. aureus* USA300) via left nostril on day seven post-boost vaccination and held upright for 1 min. Representative survival rates from two independent experiments are shown (*n* = 10). *** *p* < 0.001 for comparison with rMntC-Alum-vaccinated mice. (**C**) Mice were inoculated with 40 μL bacterial slurry (lower dose: 5 × 10^8^ CFUs of *S. aureus* USA300) as above. At 24 and 72 h, numbers of bacteria in lungs were counted (*n* = 8). (**D**) For the lower-dose (5 × 10^8^ CFUs) group, IL-1β, TNF-α, IL-6 and IL-10 were measured at 24 and 72 h (*n* = 8). (**E**) Histological evaluation of lung sections by light microscopy. Lung specimens were fixed, sectioned and stained with H&E (*n* = 6). * *p* < 0.05; ** *p* < 0.01; *** *p* < 0.001; n.s., not significant.

To evaluate the effect of EPS30 on resistance to *S. aureus* infection, we used a *S. aureus*-induced pneumonia model for comparison of EPS30 with Alum—another vaccine adjuvant. Mice were immunized twice by different combinations of subcutaneous (s.c.) and airway (intranasal, i.n.) administration routes. A total of 40 µL bacterial slurry (5 × 10^9^ CFUs of *S*. *aureus* USA300) was inoculated on day seven post-boost vaccination, and animals were monitored for up to 72 h. As shown in Figure 1B and Figure S2A,B, different methods of rMntC-EPS30 immunization resulted in different survival rates. s.c./i.n. immunization (first immunization by subcutaneous and boost immunization by intranasal) resulted in the higher survival rate (80–90%) compared with s.c/s.c immunization (twice immunization both by subcutaneous; 70%) and i.n./i.n. immunization (twice immunization both by intranasal; 40–50%) post-*S. aureus* infection. Either s.c./s.c. or s.c./i.n. immunization, survival rates of rMntC-Alum were much lower than the mice immunized with rMntC-EPS30 (Figure 1B and Figure S2A). The survival rate of EPS30-vaccinated mice did not differ significantly from that of non-vaccinated mice. In addition, another *S. aureus* antigen, rGapC, one of *S. aureus*’ surface proteins, possesses GAPDH activity and reversibly catalyzes the conversion of glyceraldehyde-3-phosphate into 1,3-bisphosphoglycerate by phosphorylation also increased survival rate when formulated with EPS30 in *S. aureus*-induced pneumonia (Figure S2C).

rMntC-EPS30-induced resistance was further evaluated by treatment with a lower dose of USA300 (5 × 10^8^ CFUs) that caused morbidity but not death, allowing assessment of bacterial load and cytokine-related lung pathology. *S. aureus* levels were zero or very low in the spleen, liver and blood (data not shown). Bacterial load in lungs at 24 and 72 h was much more strongly reduced in the rMntC-EPS30-vaccinated group than in the other three groups (Figure 1C). Lung TNF-α, IL-1β and IL-6 levels at 24 h were significantly higher for rMntC-EPS30-vaccinated mice than for other groups. However, these levels were markedly lower at 72 h in the rMntC-EPS30-vaccinated group. IL-10 level was significantly higher in rMntC-EPS30-vaccinated vs. other groups, suggesting an important role of EPS30 in reducing inflammation (Figure 1D). Lung tissues of non-vaccinated and EPS30-vaccinated mice showed red color and firm texture, whereas those of rMntC-EPS30- and rMntC-Alum-vaccinated mice showed light pink color and spongy texture (Figure S2D). At 24 h post-infection, aggregates of dark purple-stained immune cells were present in rMntC-EPS30-vaccinated mice, and lung architecture was preserved. In contrast, lung cells of non-vaccinated mice showed notable damage. At 72 h, the lung tissues of rMntC-EPS30-vaccinated mice showed clear alveolar airspaces, continuous thin alveolar walls and capillary vessels, and clear delineation of extra-alveolar vessels (Figure 1E). Pneumonocytes were intact, and cell structures were clear with inflammatory cell infiltration. In contrast, lung tissues of non-vaccinated mice displayed interstitial edema, alveolar wall thickening and notable damage to pneumonocyte morphology. Taken together, these findings indicated that rMntC-EPS30 promotes stronger resistance to *S. aureus*-induced pneumonia than does rMntC-Alum. EPS30 formulated with rMntC enhanced elimination of bacteria and also suppressed inflammation associated with *S. aureus*-induced pneumonia. Most importantly, we found that rMntC-Alum and rMntC-CFA vaccination resulted in severe local inflammation and swelling at the injection site, whereas rMntC-EPS30 vaccination caused no local inflammatory response (Figure S3), suggesting EPS30 is safer than Alum or CFA.

### 3.2. rMntC-EPS30 Vaccination Induced Superior Humoral Immune Response

**Figure 2:**
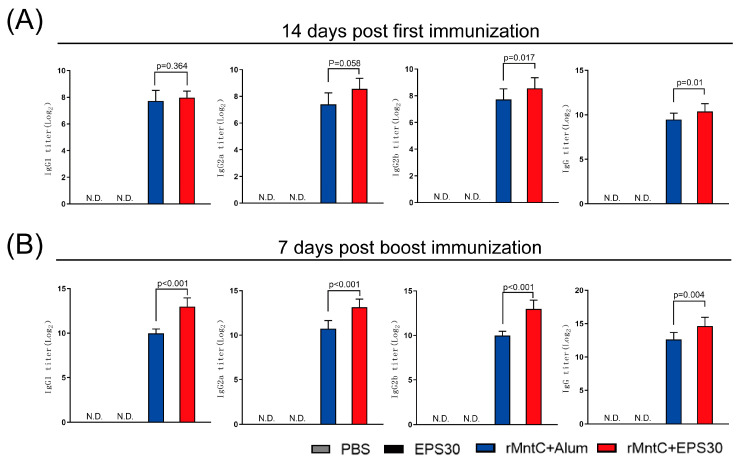
rMntC-EPS30 vaccination-induced superior humoral immune response. (**A**) Mean serum titers of rMntC-specific IgG1, IgG2a, IgG2b and IgG on days 14 post-first vaccination (*n* = 12). (**B**) Mean serum titers of rMntC-specific IgG1, IgG2a, IgG2b and IgG on days 7 post-boost vaccination (*n* = 12).

Possible mechanisms for the protective effect of rMntC-EPS30 were evaluated by examining rMntC-EPS30-induced humoral and cellular immune responses. Serum levels of rMntC-specific IgG, IgG1, IgG2a and IgG2b were measured following vaccination. Fourteen days post-first immunization, IgG and IgG2b titers were significantly higher in rMntC-EPS30-vaccinated mice than that in rMntC-Alum-vaccinated mice (Figure 2A). Seven days post-boost immunization, there were also increased levels of MntC–specific IgG, IgG1, IgG2a and IgG2b in rMntC-EPS30 vaccinated mice, significantly higher than rMntC-Alum vaccinated mice (Figure 2B). These results demonstrated that rMntC-EPS30 vaccinations could induce superior humoral immune response.

### 3.3. rMntC-EPS30 Vaccination Induced High Levels of IL-17A and IFN-γ Both in Lung and Spleen

**Figure 3:**
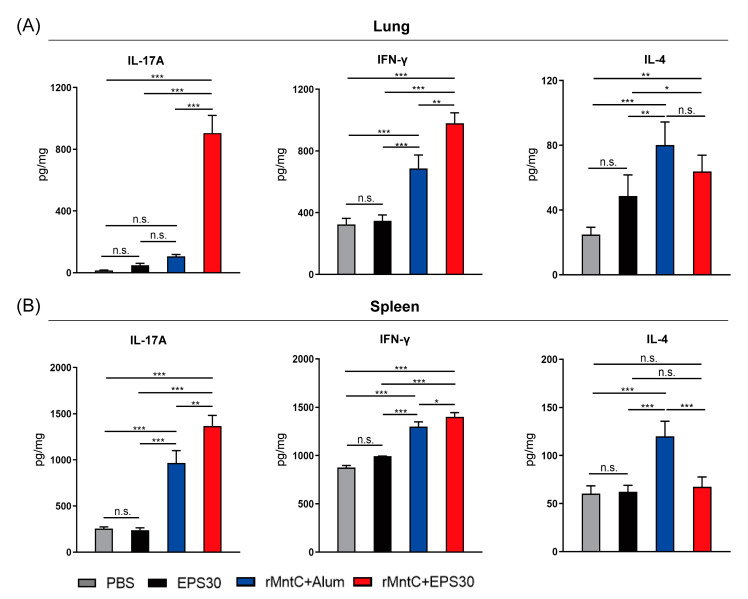
rMntC-EPS30 vaccination induced IL-17A and IFN-γ production both in the lungs and spleen. Mice were sacrificed on day seven following boost vaccination, and T cells from the lungs and spleen were prepared. (**A**) IL-17A, IFN-γ and IL-4 levels, determined by ELISA, in supernatant of lung were analyzed after 72 h rMntC-stimulation (*n* = 5). (**B**) IL-17A, IFN-γ and IL-4 levels, determined by ELISA, in supernatant of the spleen were analyzed after 72 h rMntC-stimulation (*n* = 5). * *p* < 0.05; ** *p* < 0.01; *** *p* < 0.001; n.s., not significant.

Numerous studies have demonstrated that antibodies alone are not sufficient for full protective immunity against *S. aureus*-induced diseases, and that vaccines should preferably target T cell response [31]. Accordingly, we examined effects of rMntC-EPS30 on levels of T cell-associated cytokines (IL-17A, IFN-γ, IL-4) in the lungs and spleen. On day seven post-boost vaccination, T cells of the lung and spleen were restimulated with rMntC, and secretion of these cytokines was measured. IL-17A and IFN-γ levels were much higher in the lungs and spleen of rMntC-EPS30-vaccinated mice than in rMntC-Alum-vaccinated, EPS30-vaccinated or non-vaccinated mice (Figure 3). In contrast, IL-4 level was much lower in rMntC-EPS30-vaccinated than that in rMntC-Alum-vaccinated mice. These results demonstrated that the rMntC-EPS30 vaccination could enhance IL-17A and IFN-γ levels both in the lungs and spleen.

### 3.4. rMntC-EPS Vaccination Provided Protective Effect Was Significantly Reduced in the IL-17A-Deficient Mice But Not in IFN-γ-Deficient Mice

**Figure 4:**
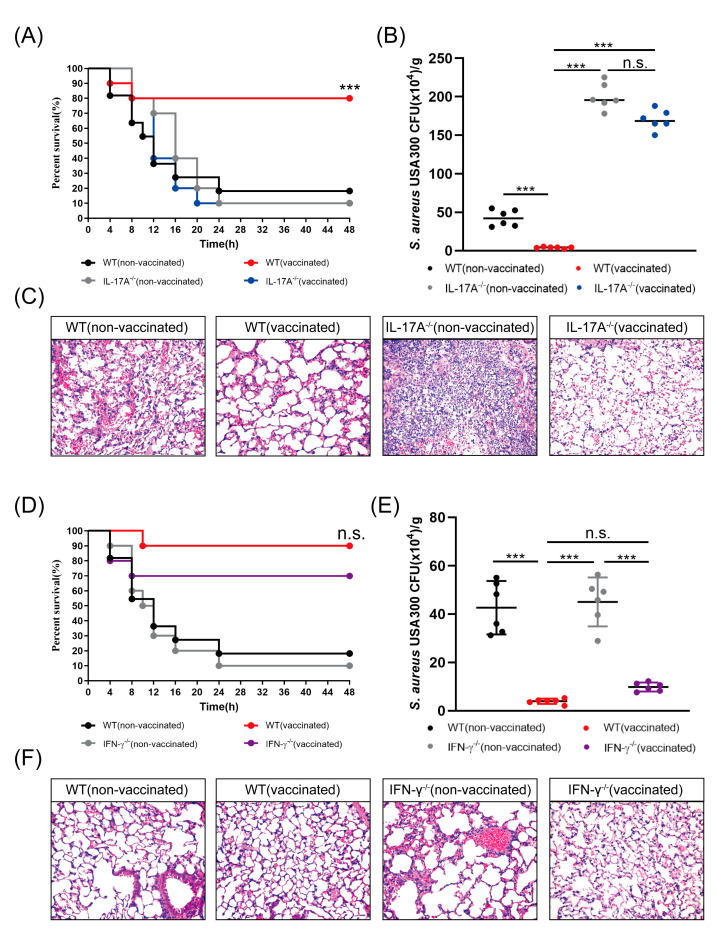
Protective effect provided by rMntC-EPS30 was significantly reduced in the IL-17A-deficient mice, but not in IFN-γ-deficient mice. (**A**) Representative survival rates of non-vaccinated and vaccinated WT and IL-17A^-/-^ mice from two independent experiments (*n* = 10). *** *p* < 0.001 for comparison with vaccinated IL-17A^-/-^ mice. (**B**) Numbers of bacteria in lungs were counted (*n* = 6) at 24 h. (**C**) Representative histological sections at 24 h (*n* = 3). (**D**) Representative survival rates of non-vaccinated and vaccinated WT and IFN-γ^-/-^ mice from two independent experiments (*n* = 10). n.s.: for comparison with vaccinated IFN-γ^-/-^ mice. (**E**) Numbers of bacteria in lungs were counted (*n* = 6) at 24 h. (**F**) Representative histological sections at 24 h (*n* = 6). *** *p* < 0.001; n.s., not significant.

Previous studies indicate that IFN-γ activates macrophages and cytotoxic T cells, and that IL-17 plays a major role in recruitment of neutrophils to *S. aureus* infection sites [12]. In view of our finding that rMntC stimulation of rMntC-EPS30-vaccinated mice enhanced IL-17A and IFN-γ production in the spleen and lung cells, we considered the possibility that rMntC-EPS30-induced production of these cytokines promoted resistance to *S. aureus*-induced pneumonia. IL-17A-deficient, IFN-γ-deficient and WT mice were vaccinated with rMntC-EPS30. Survival rate was ~80% for vaccinated WT mice, much lower (~20%) for non-vaccinated control mice and even lower (~10%) for vaccinated IL-17A-deficient mice (Figure 4A). Induced bacterial loads in the lungs post-*S. aureus* infection were higher for vaccinated IL-17A-deficient mice than for vaccinated WT mice (Figure 4B). Histological evaluation showed higher degree of lung pathology for vaccinated IL-17A-deficient mice than for vaccinated WT mice (Figure 4C).

Possible promotion of resistance to *S. aureus*-induced pneumonia by rMntC-EPS30-induced IFN-γ was examined. Survival rate was ~90% for vaccinated WT mice, somewhat lower (~70%) for vaccinated IFN-γ-deficient mice and much lower (10–20%) for non-vaccinated mice (Figure 4D). Bacterial loads in lungs at 24 h were similar for vaccinated WT and IFN-γ-deficient mice, and much higher for non-vaccinated mice (Figure 4E). Histological evaluation indicated that rMntC-EPS30 vaccination enhanced resistance for both WT and IFN-γ-deficient mice (Figure 4F). Assay for rMntC-specific IgG in serum seven days post-boost vaccination revealed no notable difference among the three groups (data not shown). These findings, taken together, indicate that the rMntC-EPS vaccine induced IL-17A, which played a substantial role in anti-*S. aureus* infection.

### 3.5. rMntC-EPS30 Vaccination Induced Robust Th17/γδ T 17 Primary and Recall Responses

**Figure 5:**
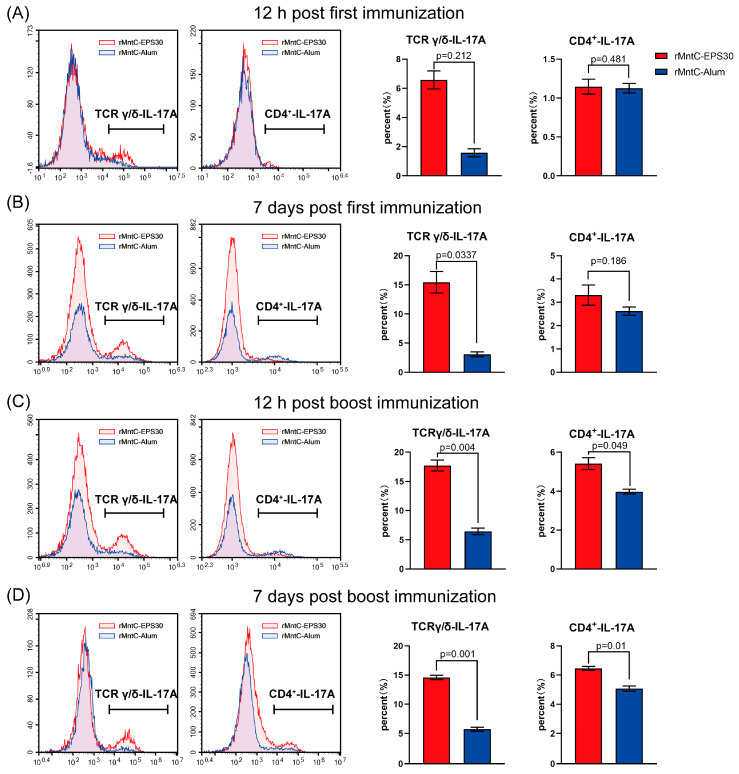
rMntC-EPS30 induced robust Th17 and γδ T17 cells responses. Analysis of production of cytokines by intracellular staining and FACS analysis. T cells isolated and purified from lung of C57BL/6 mice after the immunization. (**A**) Percentages of IL-17A-secreting γδ and CD4^+^ T cells in the lungs were detected at 12 h post-first immunization (*n* = 5). (**B**) Percentages of IL-17A-secreting γδ and CD4^+^ T cells in the lungs were detected on day seven post-first immunization (*n* = 5). (**C**) Percentages of IL-17A-secreting γδ and CD4^+^ T cells in the lungs were detected at 12 h post-boost immunization (*n* = 5). (**D**) Percentages of IL-17A-secreting γδ and CD4^+^ T cells in the lungs were detected on day seven post-boost immunization (*n* = 5).

In view of our finding (above) that resistance to *S. aureus*-induced pneumonia was promoted by rMntC-EPS30-induced IL-17A, we attempted to determine which immune cell population(s) were responsible for IL-17A production. Levels of IL-17A-secreting γδ, αβCD4^+^ and αβCD8^+^ T cells were measured at 12 h and on day seven post-every vaccination both in the lungs and spleen. Levels of IL-17A-secreting γδ T cells in lung were higher for rMntC-EPS30-vaccinated mice than for rMntC-Alum-vaccinated mice post-every immunization (Figure 5). Percentage of IL-17A-expressing γδ T cells in lung was higher (12.96–16.25%) for rMntC-EPS30-vaccinated mice than for rMntC-Alum-vaccinated (3.15–5.13%) on day seven post-boost immunization. In addition, we found that levels of IL-17A-expressing CD4^+^ T cells in rMntC-EPS30-vaccinated were significantly higher than rMntC-Alum-vaccinated mice after boost immunization (Figure 5C,D). There was no significant difference of IL-17A-expressing CD8^+^ T cells between these two groups (date not shown). In the spleen, few γδ T cells expressed IL-17A post-first immunization (Figure S4A,B). However, post-boost immunization, the level of IL-17A-expressing γδ T cells were markedly induced by rMntC-EPS30 (Figure S4C,D). The levels of IL-17A-expressing CD4^+^ T cells in mice immunized with rMntC-EPS30 were much higher than the mice immunized rMntC-Alum (Figure S4B–D). We also measured IL-17A-expressing CD8^+^ T cells, but no significant results between these two groups were observed (date not shown). These results indicated that rMntC-EPS30 vaccination could induce strong Th 17 and γδ T 17 primary T-cell responses.

**Figure 6:**
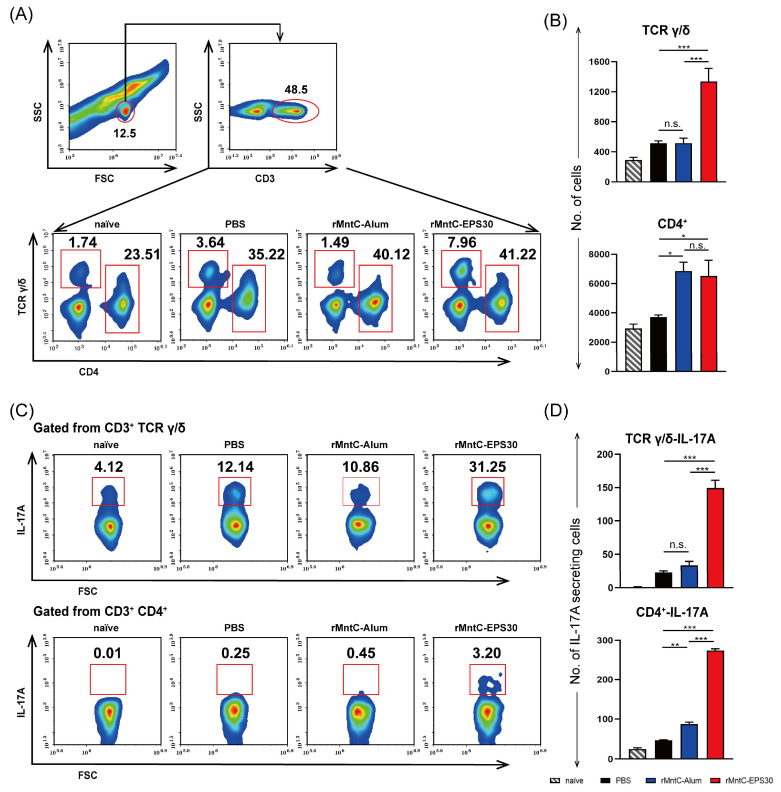
rMntC-EPS30 vaccination induced rapid and robust Th 17/γδ T 17 cells recall responses in the lung. Vaccinated mice were evaluated at 24 h post-infection. (**A**,**B**) FACS (fluorescence-activated cell sorting) analysis of the percentage (**A**) and number (**B**) of γδ T cells and CD4^+^ T cells in lungs (*n* = 5). (**C**,**D**) FACS analysis of the percentage (**C**) and number (**D**) of Th 17 cells and γδ T 17 cells in lungs (*n* = 5). * *p* < 0.05; ** *p* < 0.01; *** *p* < 0.001; n.s., not significant.

To further study the recall T-cell responses induced by rMntC-EPS30, immune responses in vaccinated mice were evaluated 24 h post-infection. Percentage and number of γδ T cells in lung were higher for rMntC-EPS30-vaccinated mice than for rMntC-Alum-vaccinated mice (Figure 6A,B). Numbers of CD4^+^ T cells in lung were similar for these two vaccinated groups, but much lower for non-vaccinated mice (Figure 6A,B). Numbers of CD8^+^ T cells post-infection were not notably elevated in either vaccinated or non-vaccinated mice (date not shown). In addition, numbers of IL-17A-secreting γδ and CD4^+^ T cells were markedly higher for rMntC-EPS30-vaccinated than for rMntC-Alum-vaccinated or non-vaccinated mice, whereas numbers of IL-17A-secreting CD8^+^ T cells did not differ notably for vaccinated vs. non-vaccinated mice (Figure 6C,D). Immune cell numbers and IL-17A production were also examined in the spleen 24 h post-infection. Findings were similar to those in lung studies (Figure S5A–D). The above results shown that rMntC-EPS30 vaccination could induce robust Th 17/γδ T 17 primary and recall responses.

### 3.6. rMntC-EPS30 Lost Both Its Increased IL-17A Secreting and Superior Protection Post-Vaccination in TCRγ/δ Knockout Mice

**Figure 7:**
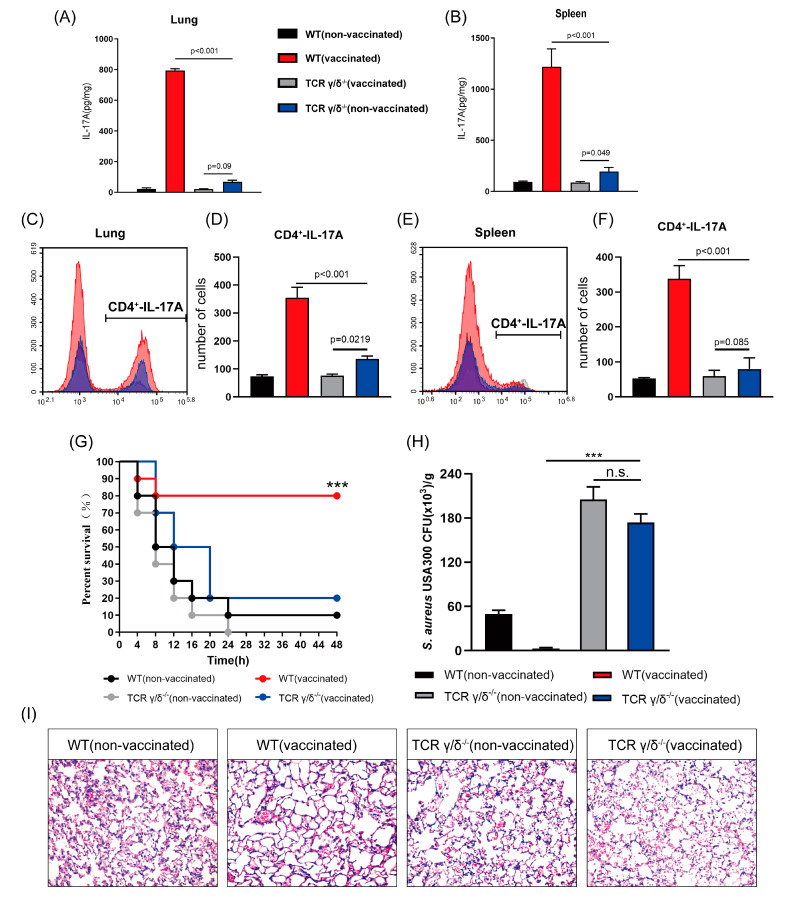
rMntC-EPS30 lost both its increased IL-17A secreting and superior protection post-vaccination in TCRγ/δ knockout mice. (**A**) IL-17A levels in supernatants of lung cells in non-vaccinated, rMntC-EPS30 vaccinated WT and γδ T cell-deficient mice, determined by ELISA on day seven post-boost vaccination (*n* = 5). (**B**) IL-17A levels in supernatants of spleen cells in non-vaccinated, rMntC-EPS30 vaccinated WT and γδ T cell-deficient mice, determined by ELISA on day seven post-boost vaccination (*n* = 5). 7 days post-boost vaccination, Th 17 cells in lungs (**C**,**D**) were assessed by flow cytometry (*n* = 5). Th 17 cells (**E**,**F**) in the spleen were assessed by flow cytometry (*n* = 5). (**G**) Representative survival rates of non-vaccinated and vaccinated WT and γδ T cell-deficient mice from two independent experiments (*n* = 10). (**H**) Numbers of bacteria in lungs were counted (*n* = 6) at 24 h. (**I**) Representative histological sections at 24 h (*n* = 6). *** *p*< 0.001; n.s., not significant.

After discovering that the effect of rMntC-EPS30 was based on induction of IL-17A-secreting γδ and CD4^+^ T cells, we focused on γδ T cells, which are generally recognized as innate immune cells, but actually play key roles in both innate and adaptive immune function [32,33]. WT and γδ T cell-deficient mice were vaccinated with rMntC-EPS30, and T cells of the lungs and spleen were prepared on day seven post-boost vaccination. T cells were re-stimulated by rMntC, and IL-17A production in culture supernatant was assayed. IL-17A production was higher in vaccinated WT mice than in vaccinated γδ T cell-deficient mice, in both the lungs and spleen (Figure 7A,B). IL-17A level in vaccinated γδ T cell-deficient mice were not significantly different from that in non-vaccinated mice. Thus, activated γδ T cells are evidently the major source of rMntC-EPS30-induced IL-17A. Percentage and number of both total CD4^+^ T cells and IL-17A-secreting CD4^+^ T cells were higher in vaccinated WT mice than in vaccinated γδ T cell-deficient mice, in both the lungs and spleen (Figure 7C–F).

The contribution of γδ T cells to *S. aureus*-induced pneumonia was evaluated by vaccinating WT and γδ T cell-deficient mice with rMntC-EPS30 and measuring survival rates in our model system. Survival rate was much higher (80%) for vaccinated WT mice than for vaccinated γδ T cell-deficient mice (20%) (Figure 7G). The number of bacteria in the lungs was significantly lower for vaccinated WT mice than for vaccinated γδ T cell-deficient mice (Figure 7H). Histological evaluation 24 h post-infection revealed complete pneumonocyte structure and substantial air space in vaccinated WT mice, whereas vaccinated γδ T cell-deficient mice showed major alveolar damage and infiltration of large numbers of immune cells (Figure 7I). These findings demonstrate that γδ T cell immune response induced by rMntC-EPS30 plays a substantial role in anti-*S. aureus*. For confirming our findings, anther model, *S. aureus* skin infection, which could also reflect the protective effect of the vaccine was used.

### 3.7. rMntC-EPS30 Vaccination Promoted Resistance to S. aureus Skin Infection

**Figure 8:**
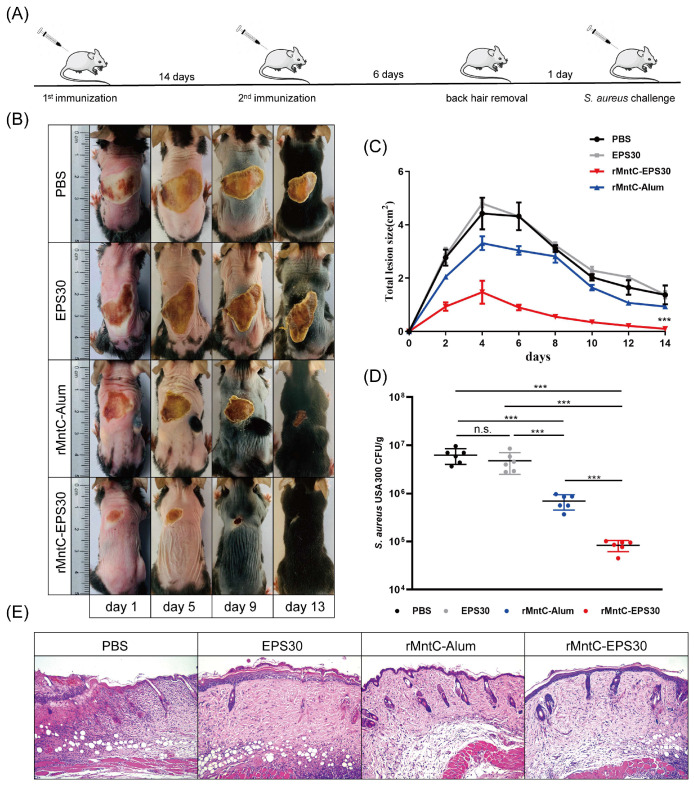
rMntC-EPS30 effectively promoted resistance to *S. aureus*-induced skin infection. (**A**) Timeline of vaccination and *S. aureus*-induced skin infection model. (**B**) Mice were dorsally s.c. injected with 50 μL bacterial slurry (5 × 10^8^ CFUs of *S. aureus* USA300) on day seven after final rMntC-EPS30 vaccination (*n* = 6). Representative photographs of skin lesions are shown. (**C**) Total lesion size (cm^2^) ± SEM (*n* = 6) are shown. *** *p* < 0.001 for comparison with rMntC-Alum-vaccinated mice. (**D**) Numbers of bacteria in lesions were counted (*n* = 6) at 24 h post-infection. (**E**) Histological evaluation of skin infection by light microscopy. Skin specimens were fixed, sectioned and stained with H&E (*n* = 6). *** *p* < 0.001; n.s., not significant.

Seven days post-boost vaccination, mice were inoculated intradermally with *S. aureus* strain USA300 (5 × 10^8^ CFUs in 50 μL saline). rMntC-EPS30-vaccinated mice developed visible skin lesions, which reached a maximal size of 1.65 ± 0.05 cm^2^ by day 4, and healed by day 13 (Figure 8B,C). rMntC-Alum-vaccinated and non-vaccinated mice developed much larger lesions, which were still not completely healed by day 21 (data not shown). Numbers of *S. aureus* CFUs in the infection site at 24 h were ~100-fold higher in non-vaccinated mice than in rMntC-EPS30-vaccinated mice (Figure 8D). Bacterial loads were much higher in rMntC-Alum-vaccinated than in rMntC-EPS30-vaccinated mice. Histological evaluation (H&E staining) of skin at 24 h revealed large neutrophilic abscesses in rMntC-EPS30-vaccinated mice (Figure 8E). Thus, resistance to *S. aureus*-induced skin infection was much more strongly promoted by rMntC-EPS30 vaccination than by rMntC-Alum vaccination.

**Figure 9:**
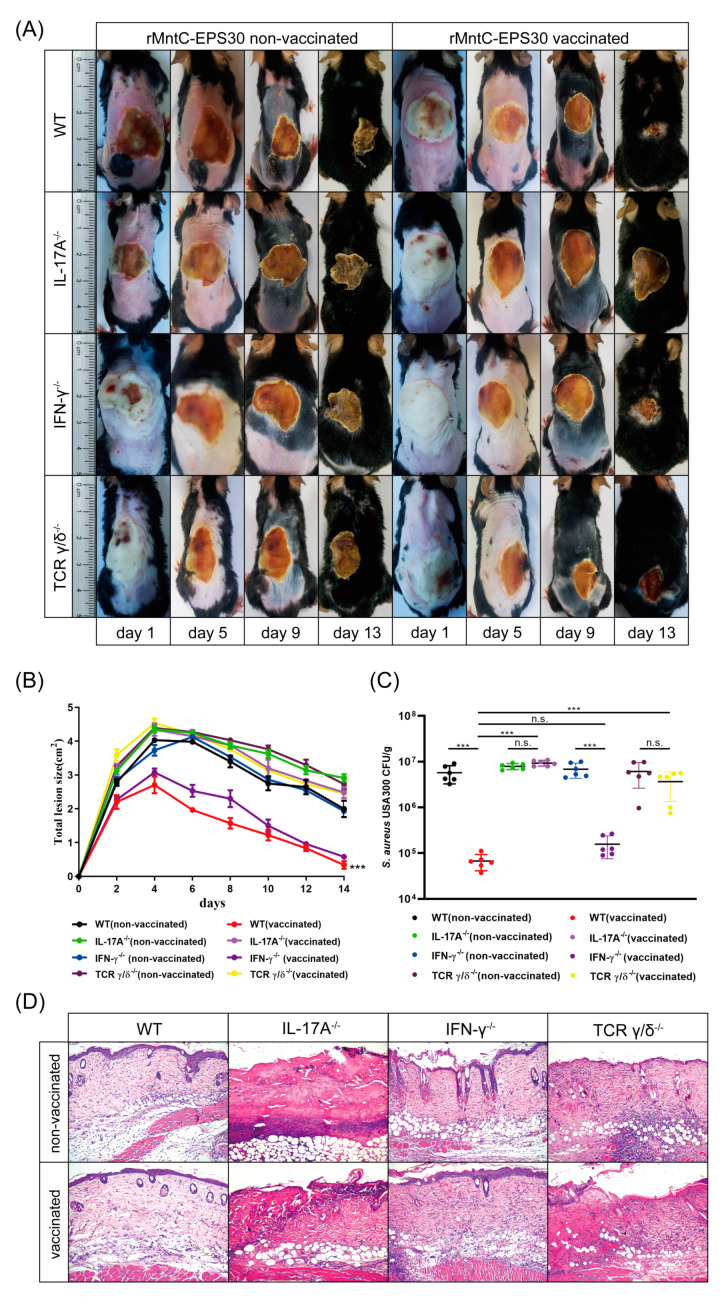
rMntC-EPS vaccine efficiency of anti-*S. aureus* skin infection was significantly reduced in the IL-17A-deficient and γδ T -deficient mice not in IFN-γ-deficient mice. (**A**) Mice were dorsally s.c. injected with 50 μL bacterial slurry (5 × 10^8^ CFUs of *S. aureus* USA300) on day seven after final rMntC-EPS30 vaccination (*n* = 6). Representative photographs of skin lesions. (**B**) Total lesion size (cm^2^) ± SEM (*n* = 6). *** *p* < 0.001 for comparison with vaccinated IL-17A-deficient and γδ T cell-deficient mice. (**C**) Numbers of bacteria in lesions were counted (*n* = 6) at 24 h. (**D**) Representative histological sections at 24 h (*n* = 6). *** *p* < 0.001; n.s., not significant.

To confirm the above findings, IL-17A-deficient, IFN-γ-deficient and γδ T cell-deficient mice were inoculated intradermally with *S. aureus* strain USA300 on day seven post-boost vaccination. Vaccinated WT and IFN-γ-deficient mice developed visible skin lesions (Figure 9A,B) and reduced bacterial loads (Figure 9C), to similar degrees. In contrast, vaccinated IL-17A-deficient and γδ T cell-deficient mice developed lesions which were ~2-fold larger and did not heal completely by day 21. Numbers of *S. aureus* CFUs on day one were ~100-fold higher in vaccinated IL-17A-deficient and γδ T cell-deficient mice than in vaccinated WT and IFN-γ-deficient mice. Histological evaluation revealed cutaneous inflammation with neutrophil infiltration in vaccinated WT and IFN-γ-deficient mice; in contrast, skin of vaccinated IL-17A-deficient and γδ T cell-deficient mice showed major damage (Figure 9D). These findings, taken together, indicated that the rMntC-EPS vaccine induced γδ T cells and IL-17A play substantial roles in anti-*S. aureus* immunity.

## 4. Discussion

*Staphylococcus aureus* is a serious pathogen that causes pneumonia in humans and other mammals, resulting in high mortality rates [2]. *S*. *aureus*-induced infections are difficult to treat because of their complex pathogenic mechanisms, and the ability of this bacterium to quickly become resistant to various antibiotics [34,35]. Numerous attempts have been made during the past two decades to develop an effective *S. aureus* vaccine, but success to date is quite limited. Increasing evidence indicates that the pro-inflammatory cytokine IL-17 in mice is essential for host defense against cutaneous *S. aureus* infection [11,20]. Th17 cells were previously thought to be the sole producers of IL-17 during *S. aureus* infection; however, γδ T cells have recently been implicated as the major source of IL-17 production (~80% of IL-17-secreting CD3^+^ T cells) [20]. Here, we developed a vaccine, termed rMntC-EPS30, composed of *S. aureus* antigen MntC and exopolysaccharides derived from probiotic *L. casei*, which significantly increased survival rate in *S. aureus*-induced pneumonia cases and reduced the size of lesions in *S. aureus*-induced skin infection in mice. Using mouse pulmonary and cutaneous models, we demonstrated that γδ T cells and IL-17A induced by rMntC-EPS30 may promote resistance to *S. aureus* infection.

Adjuvants activate innate immune system and also generate cytokine milieus that modulate vaccine-induced Th cell differentiation [21]. The type of vaccine-induced immune response is greatly affected by administration route and selection of adjuvant [36]. Alum is the most widely used adjuvant in human and animal vaccines, and was recently shown to promote IL-1β secretion through NLRP3 activation. Alum effectively enhances humoral immune response, but has little effect on cellular immune response [37]. Studies of numerous mouse infection models have demonstrated that antibodies alone are not sufficient to produce complete immunity to various *S. aureus*-induced diseases. Next-generation vaccine design must be based on the targeting of T cell responses. Exopolysaccharides produced by lactobacilli are beneficial in many ways for human and animal health; these include antitumor, anti-inflammatory, anti-ulcer, anti-cancer, antioxidant and immune-modulating activities. We demonstrated previously that EPS30 has adjuvant properties in vivo and in vitro, and upregulates IFN-γ and IL-17 expression in CD4^+^ T cells [25]. We therefore proposed that formulation of EPS with *S. aureus* antigen as an adjuvant might be provide effective protection against *S. aureus* infection. In our *S. aureus*-induced pneumonia model, survival rate of rMntC-EPS30-vaccinated animals was up to 90% by s.c./i.n. immunization and significantly higher than that of rMntC-Alum-vaccinated animals (Figure 1B). EPS30 was likewise a more effective adjuvant than Alum in our *S. aureus* skin infection model (Figure 8B–E).

Vaccine development has traditionally focused on stimulating antibody response. Anti-*S. aureus* antibodies play important roles in blocking toxins involved in immune cell lysis, and in providing opsonic help to phagocytes [38]. However, phase III clinical trials of two distinct vaccine candidates did not show efficacy in protection against bacteremia because of overreliance on antibody-mediated protective effect, suggesting that vaccines targeting T cell response may be preferable [5]. Conventional CD4^+^ T cells have been shown to be essential for anti-*S. aureus* responses in several mouse infection models, and in humans. However, vaccines designed to target T cell responses (SA3Ag, SA4Ag and Pentavalent vaccines) still displayed limited success in clinical trials [39,40,41]. CD4^+^ T cells, and production of IFN-γ and IL-17 by these cells, have been investigated extensively in regard to *S. aureus* infection, and are considered promising vaccine targets. However, CD4^+^ T cells are not the sole producers of these cytokines. Several “alternative” lymphoid cell types have been shown to have functions overlapping those of conventional T cells. Recent studies suggest an important role of these alternative lymphoid cells in infection response. These cells, which include γδ T cells, innate lymphoid cells (ILCs) and mucosal-associated invariant T (MAIT) cells, display properties of both innate and adaptive immune responses and importantly, in this context, produce cytokines (IFN-γ, IL-4, IL-17, and others) involved in responses to bacterial infections.

γδ T cells are major histocompatibility complex (MHC)-unrestricted cells that comprise a small proportion of the overall lymphoid population but are abundant at barrier sites such as skin, lungs and adipose tissue. In general, differing γδ T cell subsets are associated with specific tissues. γδ T cells evidently play a key role in immune response to *S. aureus* infection, at least in mice [15,19]. The ability of these cells to produce IL-17 is of particular interest. Th17 cells were previously thought to be the sole producers of IL-17 during *S. aureus* infection; however, γδ T cells have recently been implicated as the major source of IL-17 production (~80% of IL-17-secreting CD3^+^ T cells) [18]. Following peritoneal inoculation of mice with *S. aureus*, γδ T cells were the primary IL-17 producers during the initial 3 h. Similar rapid production of IL-17 by γδ T cells was observed in a s.c. infection model, a surgical wound infection model, and in intranasally infected mice [15,16]. In γδ T cell-deficient mice, neutrophil recruitment to infection site was greatly reduced, and γδ T cells functioned as the primary effectors to limit bacterial load. Using a pneumonia model, Q. Zou’s group demonstrated that γδ T cell-deficient mice had >100-fold greater bacterial load in lungs, and findings in a surgical wound infection model were similar, indicating an essential role of γδ T cells in infection control. Immunomodulatory therapy or development of vaccines to induce IL-17-secreting γδ T cells therefore have strong potential for improved treatment of *S. aureus* infections.

Our findings indicate that γδ T cells and IL-17A induced by rMntC-EPS30 promotes resistance to *S. aureus* infection. Levels of rMntC-specific IL-17A in both the spleen and lungs was significantly lower in rMntC-EPS30-vaccinated γδ T cell-deficient mice than in vaccinated WT mice (Figure 7A,B). In models of *S. aureus*-induced pneumonia and skin infection, rMntC-EPS30 vaccination strongly promoted infection resistance in WT mice, but had no effect in γδ T cell-deficient mice (Figure 7G and Figure 9A).

In summary, a newly developed vaccine, rMntC-EPS30, composed of *S. aureus* antigen MntC and a novel adjuvant EPS30, provided effective protection against *S. aureus*-induced pneumonia and skin infection, and is a promising candidate for future clinical application.

## 5. Conclusions

In this study, we developed a novel vaccine (termed rMntC-EPS) composed of *S. aureus* antigen MntC and *Lactobacillus casei* exopolysaccharides, which conferred up to 90% protection against *S. aureus* pulmonary infection and induced strong protection against *S. aureus* cutaneous infection. We demonstrated that the rMntC-EPS vaccine induced γδ T cells and IL-17A might play substantial roles in anti-*S. aureus* immunity. In addition, we found that rMntC-Alum and rMntC-CFA vaccinations resulted in severe local inflammation and swelling at the injection site, whereas the rMntC-EPS30 vaccination caused no local inflammatory response, suggesting that EPS30 is safer than Alum or CFA. Our findings provided direct evidence that the rMntC-EPS vaccine is a promising candidate for future clinical application against *S. aureus*-induced infections.

## Data Availability

All data that this study is based upon are available from the corresponding authors upon request.

## Supplementary Materials

The following are available online at https://www.mdpi.com/article/10.3390/vaccines9070775/s1, Figure S1: Cloning, expression, purification, and evaluation of recombinant MntC, Figure S2: rMntC-EPS30 conferred effective protection against *S. aureus*-induced pneumonia. Figure S3: EPS30 is safer than Alum or CFA. Figure S4: rMntC-EPS30 induced robust Th17 and γδ T17 cells primary responses. Figure S5: rMntC-EPS30 vaccination induced rapid and robust Th 17/γδ T 17 cells recall responses in the spleen.
