# Supplementary figures and images for: Vaccine Composition Formulated with a Novel Lactobacillus-Derived Exopolysaccharides Adjuvant Provided High Protection against Staphylococcus aureus
Source: Vaccines (Basel). 2021 Jul 12;9(7):775. doi: 10.3390/vaccines9070775 (PMC8310297; doi:10.3390/vaccines9070775)

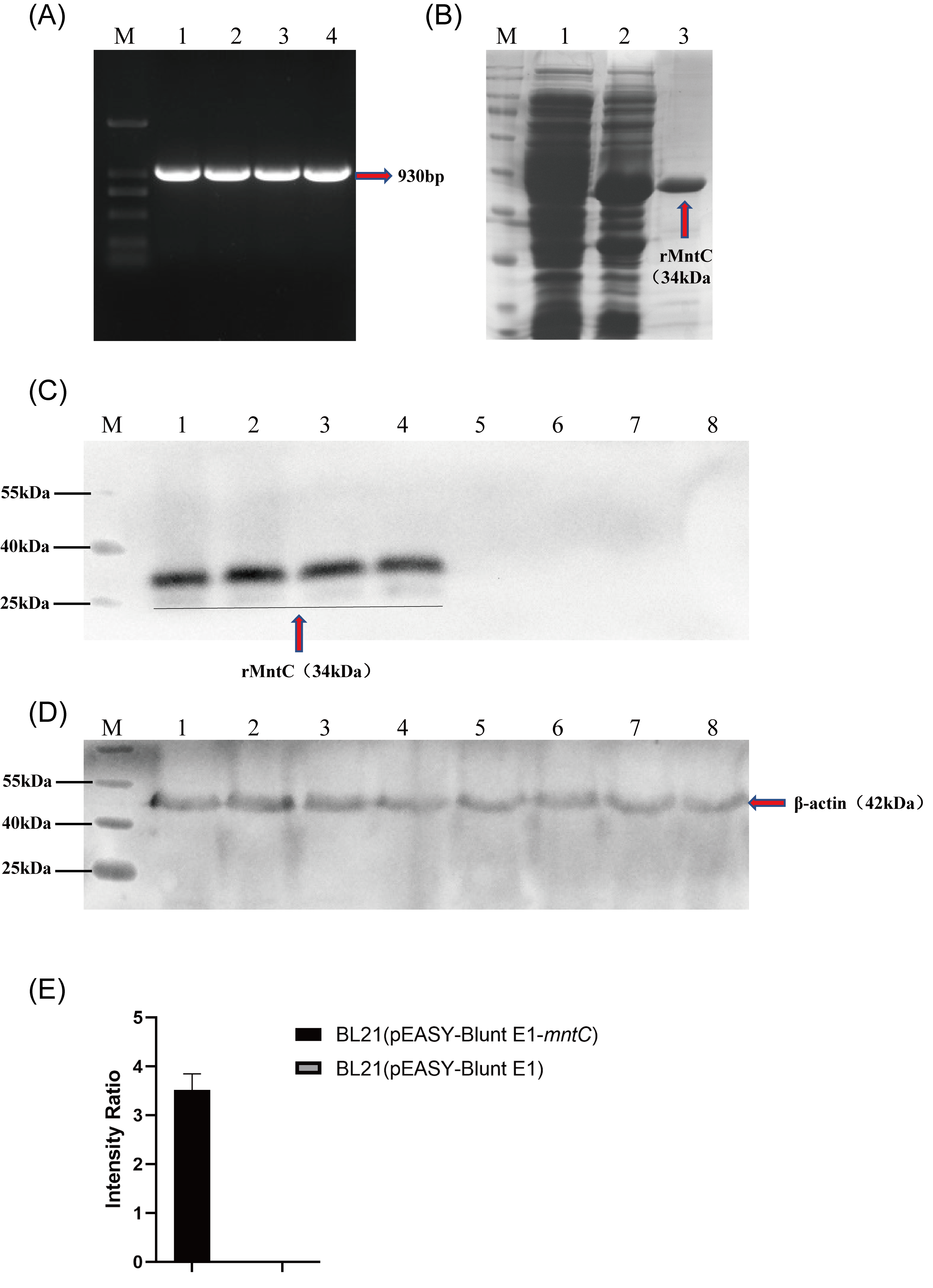

Supplement: Supplementary file 1 [file vaccines-09-00775-s001.zip › Supplementary Fig. 1.tif]

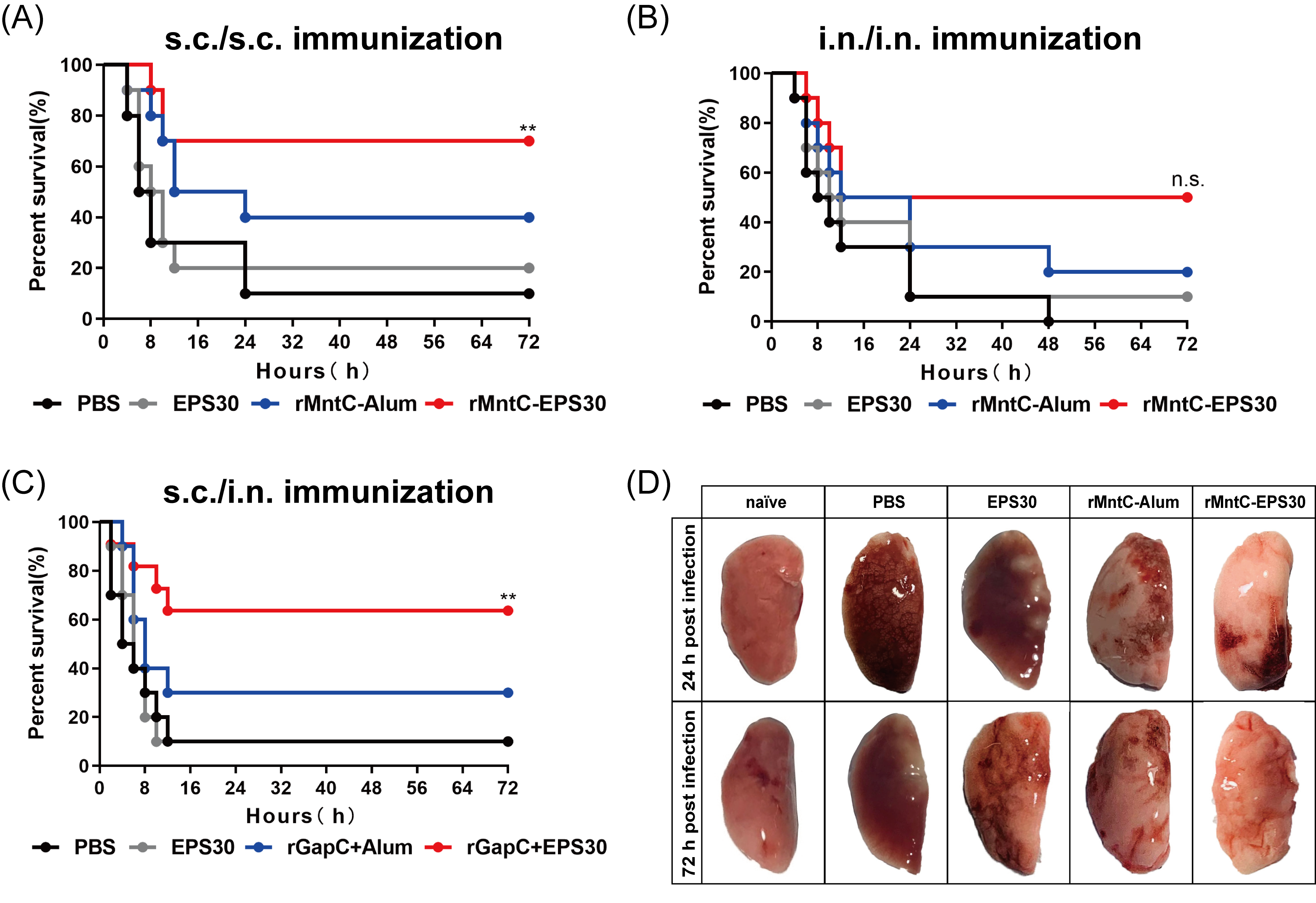

Supplement: Supplementary file 1 [file vaccines-09-00775-s001.zip › Supplementary Fig. 2.tif]

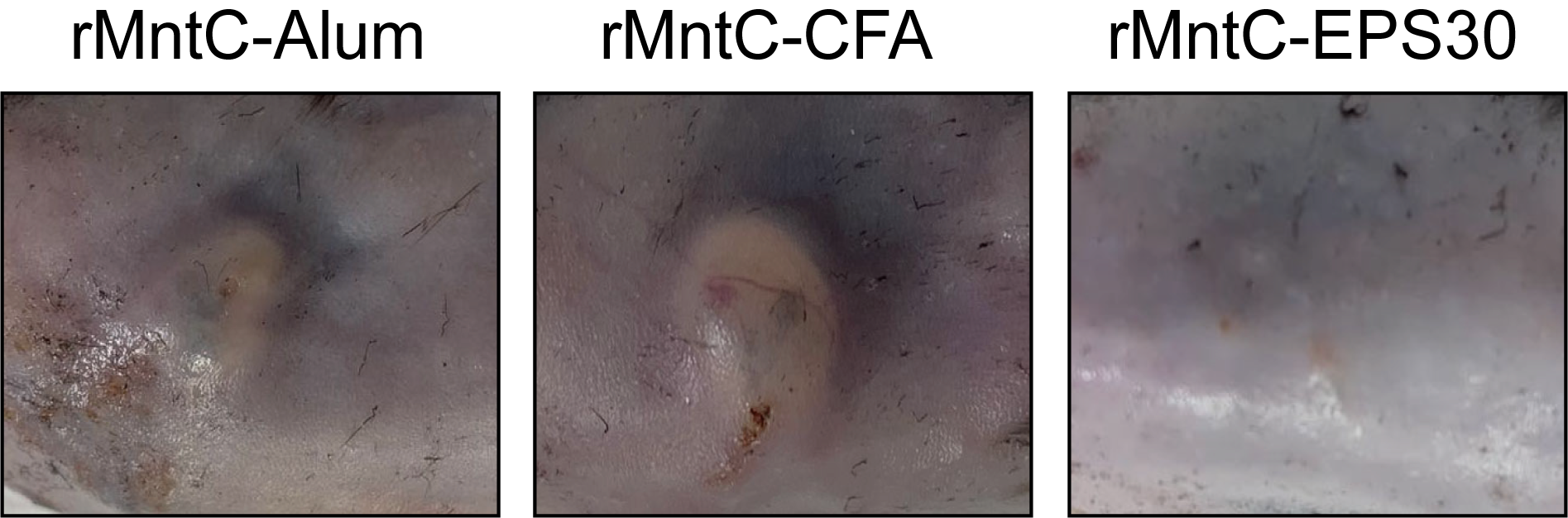

Supplement: Supplementary file 1 [file vaccines-09-00775-s001.zip › Supplementary Fig. 3.tif]

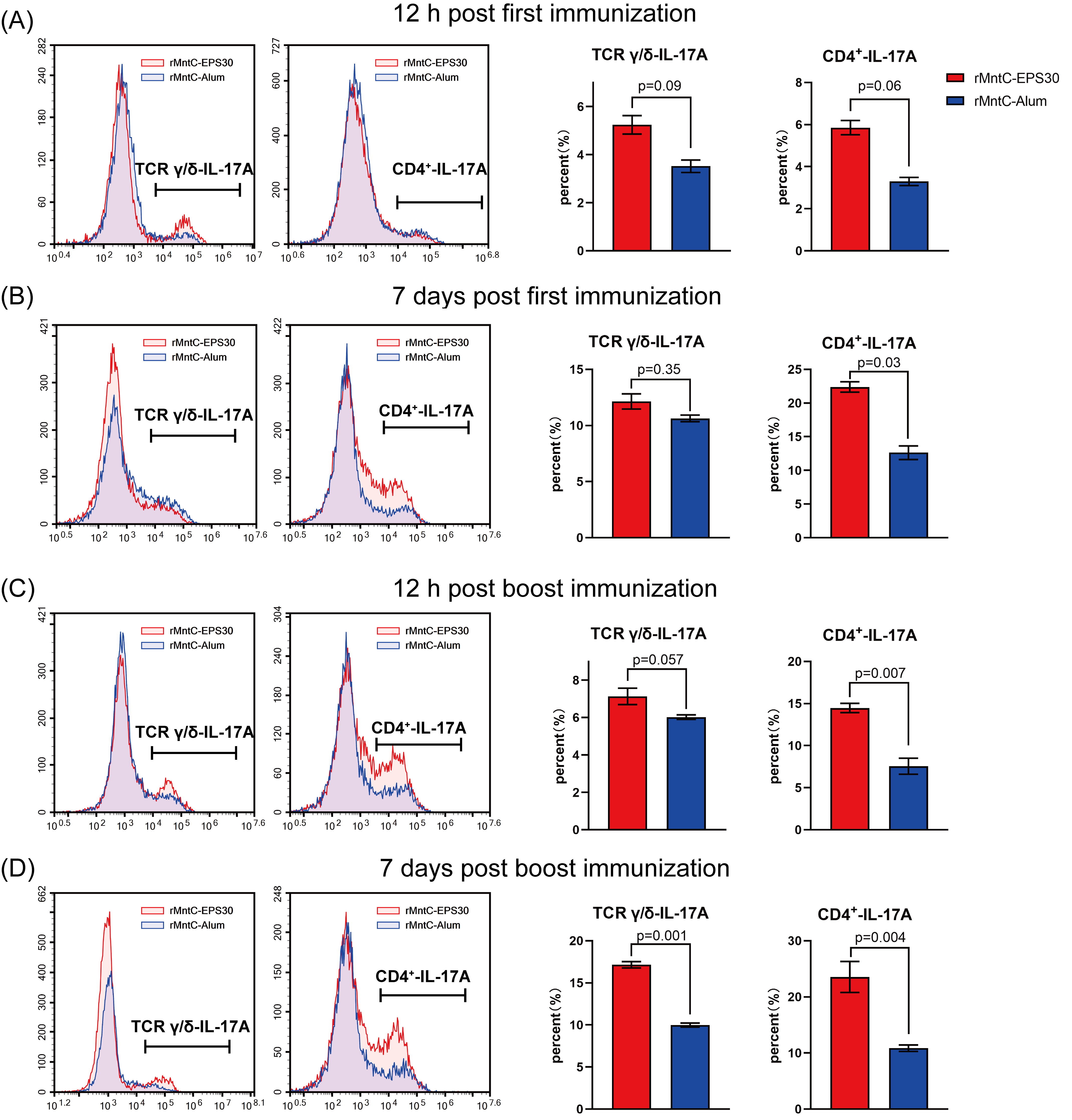

Supplement: Supplementary file 1 [file vaccines-09-00775-s001.zip › Supplementary Fig. 4.tif]

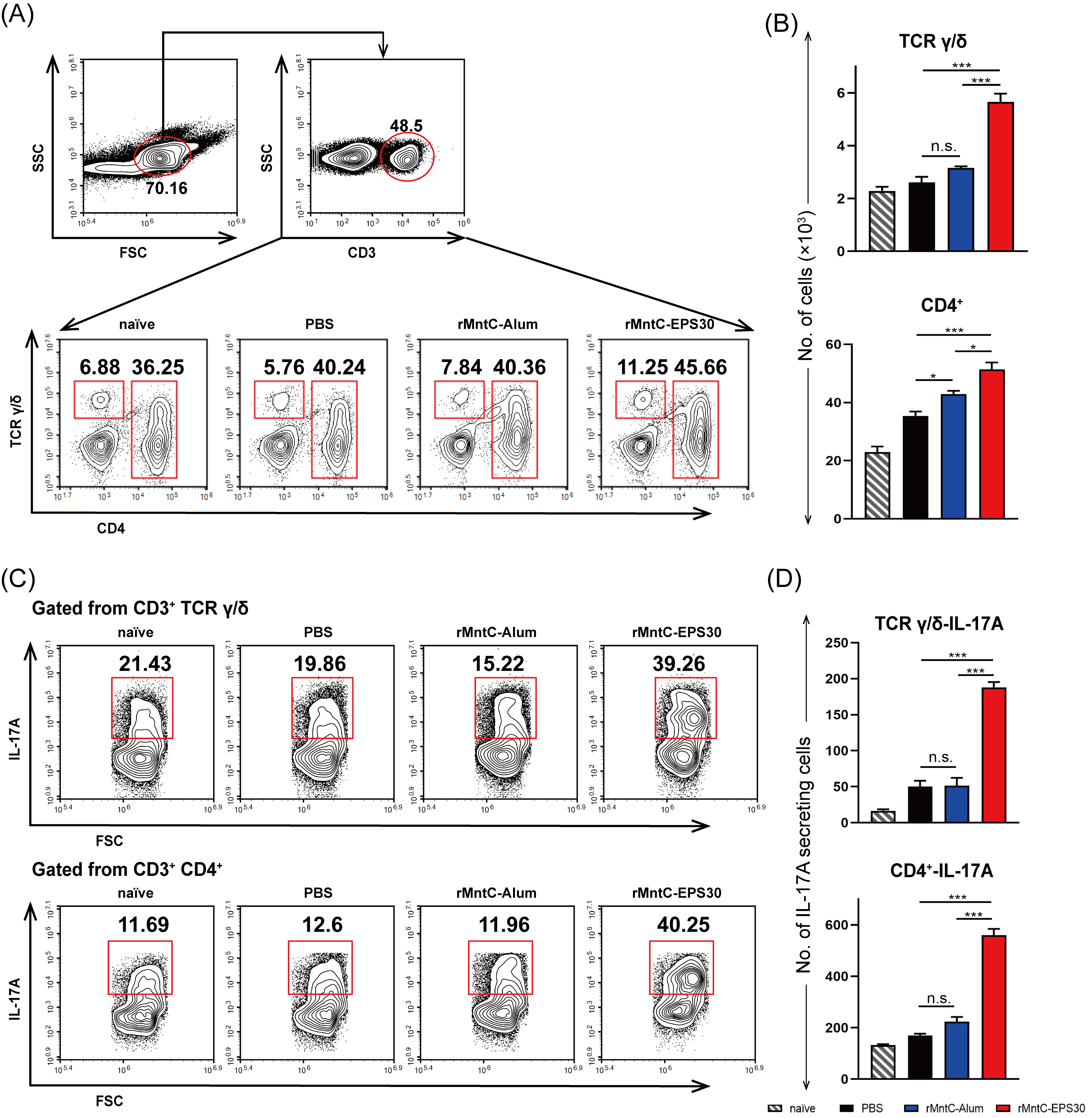

Supplement: Supplementary file 1 [file vaccines-09-00775-s001.zip › Supplementary Fig. 5.tif]
